# Bone tissue engineering via growth factor delivery: from scaffolds to complex matrices

**DOI:** 10.1093/rb/rby013

**Published:** 2018-06-09

**Authors:** Tinke-Marie De Witte, Lidy E Fratila-Apachitei, Amir A Zadpoor, Nicholas A Peppas

**Affiliations:** 1 Department of Biomedical Engineering, The University of Texas at Austin, Austin, TX, USA; 2 Additive Manufacturing Laboratory, Department of Biomechanical Engineering, Delft University of Technology (TU Delft), CD Delft, The Netherlands; 3 McKetta Department of Chemical Engineering, The University of Texas at Austin, Austin, TX, USA; 4 Institute for Biomaterials, Drug Delivery and Regenerative Medicine, The University of Texas at Austin, Austin, TX, USA; 5 Department of Pediatrics, and Department of Surgery and Perioperative Care, Dell Medical School, The University of Texas at Austin, Austin, TX, USA; 6 Division of Molecular Pharmaceutics and Drug Delivery, College of Pharmacy, The University of Texas at Austin, Austin, TX, USA

**Keywords:** scaffolds, bone growth, tissue engineering, growth factor delivery

## Abstract

In recent years, bone tissue engineering has emerged as a promising solution to the limitations of current gold standard treatment options for bone related-disorders such as bone grafts. Bone tissue engineering provides a scaffold design that mimics the extracellular matrix, providing an architecture that guides the natural bone regeneration process. During this period, a new generation of bone tissue engineering scaffolds has been designed and characterized that explores the incorporation of signaling molecules in order to enhance cell recruitment and ingress into the scaffold, as well as osteogenic differentiation and angiogenesis, each of which is crucial to successful bone regeneration. Here, we outline and critically analyze key characteristics of successful bone tissue engineering scaffolds. We also explore candidate materials used to fabricate these scaffolds. Different growth factors involved in the highly coordinated process of bone repair are discussed, and the key requirements of a growth factor delivery system are described. Finally, we concentrate on an analysis of scaffold-based growth factor delivery strategies found in the recent literature. In particular, the incorporation of two-phase systems consisting of growth factor-loaded nanoparticles embedded into scaffolds shows great promise, both by providing sustained release over a therapeutically relevant timeframe and the potential to sequentially deliver multiple growth factors.

## Introduction

Bone acts as the supportive structure of the body, functions as a mineral reservoir, guards vital organs, is the site of blood cell production and helps maintain acid–base balance in the body [1]. The importance of bone is reflected by the staggering economic and clinical impact of bone defect treatment as a result of diseases such as osteogenesis imperfecta, osteoarthritis, osteomyelitis and osteoporosis [2].

Indeed, the ageing population paired with an increase in obesity and poor physical activity has led to an increase in the occurrence of bone disorders that include bone fractures, low back pain, scoliosis, osteoporosis, bone infection, tumors and rheumatic diseases [3, 4]. In fact, more than 20 million people annually worldwide are affected by a loss of bone tissue caused by trauma or disease [5], and in the USA alone, over half a million bone defect repairs occur annually representing over $2.5 billion in costs [3].

While bone is known for its ability to self-heal, large-scale defects greater than a critical size hinder the natural bone-healing process and do not allow for complete fracture healing [6]. These large bone defects, or non-unions, can occur as the result of traumatic injury, tumor resections, or congenital defects and highly correlate with factors including severity of injury, extent of soft tissue damage, advanced age and comorbidities such as diabetes [7, 8].

Common treatments for critical-size bone defects include bone autografts and allografts. Indeed, of the 20 million people affected by a lack of bone tissue annually worldwide, about 5 million cases require orthopedic intervention [5], with grafting procedures representing about 60% of these interventions [9]. However, these treatment approaches present serious limitations in the repair and regeneration of bone.

The current gold standard in critical-size bone fracture repair is the use of autologous bone grafts (autografts). Autografts require harvesting bone from the patient’s iliac crest and transplanting to the fracture site. The key advantages of this approach include the fact that autologous bone grafts are histocompatible and non-immunogenic [3]. In fact, autografts present growth factors (GFs), osteoprogenitor cells and a three-dimensional (3D) matrix, which are essential components for osteoinduction, osteogenesis and osteoconduction, respectively. However, autografts require a second operation at the site of harvest and are therefore expensive and present surgical risks such as bleeding, inflammation, infection and chronic pain, as well as donor site injury and morbidity, deformity, hypersensitivity and scarring [10].

In addition, due to limited sources, autografts may not be an option when dealing with very large defects [11]. Allogeneic grafts (allografts) are the second most common treatment option for large bone defects and involve transplanting donor bone tissue, often from a cadaver. This method also faces limitations such as shortage of donors, high cost, need for sterilization and activation and risk of viral disease transmission, bacterial infection or immune rejection [12]. The limitations and disadvantages associated with auto- and allograft harvesting point to the clinical need for alternative bone repair strategies. This has led to the development of bone tissue regeneration approaches such as bone tissue engineering [13].

The aim of bone tissue engineering is to develop 3D scaffolds that mimic the extracellular matrix (ECM) and provide mechanical support thereby aiding in the formation of new bone. Scaffolds provide a template for cell attachment and stimulate functional bone tissue formation *in vivo* through tailored biophysical cues to direct the organization and behavior of cells [6, 14]. These scaffolds are designed to be chemically biocompatible, biodegradable, and porous such that they promote vascularization, have sufficient mechanical strength and provide physical and biochemical stimuli [15]. The advantages of these acellular scaffolds for bone tissue engineering include the ease of sterilization, longer shelf-lives and low potential for infection or immunogenicity [2].

A new generation of tissue engineering scaffolds seeks to further improve tissue regeneration through the local delivery of bioactive molecules crucial to the natural formation of bone. Specifically, GFs play a key role in tissue regeneration and have led to a new GF-based strategy to improve the tissue-healing process [16]. However, a key limitation to GF therapy for tissue regeneration is the need for large quantities due to the fast inactivation and clearance of GFs. This leads to the need for supraphysiological levels that are associated with high treatment costs and high risk of adverse effects [7]. New strategies are therefore geared toward the local, targeted delivery of bioactive molecules crucial to natural bone regeneration through scaffold-based approaches. These scaffolds could then retain the biomolecules at the fracture site *in vivo* and reduce the required dose of GFs [13]. These GFs then act to recruit endogenous stem cells from adjacent tissues and direct their differentiation into bone tissue within the porous scaffold matrix [17].

The aim of this review is to outline the current directions of scaffold-based GF delivery research. We outline the different requirements and materials required in the fabrication of a scaffold for bone tissue engineering. We then evaluate the different GFs involved in bone regeneration and explore the different scaffold-based GF delivery approaches found in the recent literature.

## Bone tissue engineering scaffolds

### Scaffold requirements

**Figure 1.**
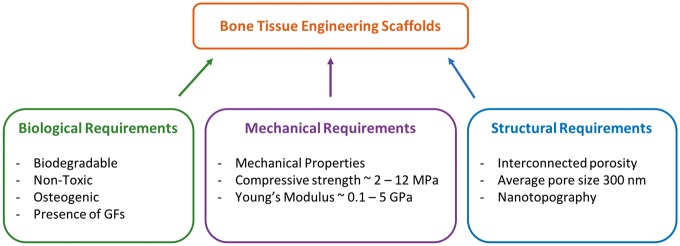
Biological, mechanical, and structural requirements for an ideal bone tissue engineering scaffold

Bone tissue engineering scaffolds are 3D structures that provide an architecture and environment for bone tissue to develop and grow, guiding the spatially and temporally complex process of bone fracture repair as reviewed by Hankenson *et al.* [18]. Indeed, scaffolds are designed to promote cell adhesion, survival, migration and proliferation, accelerate bone remodeling, provide osteoconductive structural guidance, and in some cases act as carrier materials for GFs, antibiotics or gene therapy [1, 19]. A successful bone tissue engineering system must include: (i) a chemically and mechanically biocompatible scaffold that mimics the ECM; (ii) the presence of morphogenic signals to recruit and direct osteogenic cells; and (iii) vascularization to provide nutrient supply for the new tissue [3]. In order to satisfy these components, a successful scaffold must meet certain biological, mechanical and structural requirements (Fig. 1).

Biological requirements are that the scaffold must be cytocompatible, biodegradable and non-toxic [4]. Indeed, the scaffold determines the anatomical form of the bone to be regenerated and over time as bone-healing progresses, cells invade and deposit new bone matrix. The scaffold is designed to degrade as the bone-regeneration process advances, leaving behind only the newly formed functional tissue. Biocompatibility is also crucial as the clinical success of the scaffold is highly reliant on positive interactions with the adjacent tissue structures [19].

In addition to these biological requirements, a successful scaffold for bone tissue engineering applications must meet certain mechanical requirements. Scaffold mechanical properties should be tailored to match those of the host tissue in order to reduce the chance of complications such as post-operation stress shielding, implant-related osteopenia or subsequent refracture [3, 10]. Typically, scaffolds are designed to match the mechanical properties of human cancellous bone which has a compressive strength between 2 and 12 MPa and an elastic modulus between 0.1 and 5 GPa [10].

While a scaffold must have sufficient mechanical properties for optimal performance, it must also present a degree of porosity that is essential to proper cell growth and migration, nutrient flow, vascularization and spatial organization [3]. In fact, it has been shown that scaffolds with a minimum pore size of 150 μm and a mean pore size of 300 μm are optimal for bone tissue formation [6]. It is believed that a porous structure with a mean pore size greater than 300 μm promotes angiogenesis thereby improving bone regeneration. The porous structure of the scaffold is also essential for osseointegration with the host bone as it allows for interlocking between the scaffold and the surrounding tissue, ensuring long-term stable fixation of the implanted scaffold [10, 15]. In addition to pore size, pore interconnectivity is a key requirement. Indeed, interconnected porosity is important for continuous bone ingrowth as well as for waste removal and nutrient transport toward the center of the scaffold [6, 20]. The presence of an open and interconnected porous network is also essential for proper tissue vascularization. However, a key challenge in the fabrication of bone tissue engineering scaffolds is to develop a mechanically strong scaffold with sufficient porosity so as to retain proper vascularization.

An additional structural requirement is the incorporation of nanotopographic characteristics. Indeed, the presence of nanotopography has been shown to influence the osteoinductivity and osteointegration of scaffolds for bone tissue engineering [3]. These structural features can be included in the form of nanopatterns, nanopores or surface topography [4]. Ultimately, one of the main challenges in the design of scaffolds for bone tissue engineering applications is the incorporation of a micro- and nanoscale dimensional hierarchy representative of native bone [2]. One promising method to achieve such hierarchical structures is through the development of self-folding, ornamented flat constructs to form 3D lattices with desired nanotopography [21].

### Scaffold materials

In order to meet the biological, mechanical and structural requirements for a successful bone tissue engineering scaffolds, different categories of materials have been explored. Metals, ceramics, polymers and their composites have been studied for their osteogenic properties and ability to support the formation of new, functional bone. These materials present characteristic advantages and limitations as evidenced by their *in vitro* and *in vivo* biocompatibility and osteogenicity. This wide variety of materials also presents a wide range of scaffold fabrication techniques including gas foaming, solvent casting, particle leaching, freeze drying, thermally induced phase separation, foam gel and 3D printing [6]. A summary of these different materials and their key advantages and limitations is provided in Table 1.

**Table 1.** Summary of materials and techniques used to fabricate bone tissue engineering scaffolds and their main advantages and limitations

Scaffold material	Examples	Fabrication methods	Advantages (+) and limitations (−)	References
Metals	NiTi, titanium alloy, magnesium alloy, porous tantalum	3D Printing, casting, powder sintering	+ High young’s modulus + High compressive strength − Not degradable − Ion release	[22–25]
Ceramics	TiO_2_, HAp, β-TCP, Bioglass	3D Printing, sol-gel, selective laser sintering	+ Chemically biocompatible + Can be biodegradable − Brittle − Prone to fracture and fatigue	[26–30]
Natural polymers	Collagen, chitosan, hyaluronic acid, silk fibroin	Hydrogel crosslinking, electrospinning, freeze drying, solvent displacement	+ Biocompatible + Biodegradable + Osteogenic − Low mechanical strength	[31–33]
Synthetic polymers	PLGA, PCL, PEO, PPF	Electrospinning, crosslinking	+ Tunable properties − Acidic degradation byproducts − Rapid strength degradation in vivo	[14, 34, 35]

HAp: Hydroxyapatite; b-TCP: beta-Tricalcium Phosphate; PLGA: poly(D,L-lactic-glycolic acid); PCL: polycaprolactone; PEO: poly(ethylene oxide); PPF: poly(propylene fumarate)

#### Metals

Metals have historically been used as biomaterials for various bone-related purposes, thanks to their mechanical strength specifically in load-bearing applications. Indeed, implants made from metals such as titanium, magnesium or stainless steel have been used for joint prostheses, plates and screws [4]. In recent years, metals have also been explored for their ability to be fabricated into porous scaffolds to support the regeneration of bone tissue. For example, Van Bael *et al.* developed selective laser-melted Ti6Al4V scaffolds capable of promoting the adhesion and differentiation of osteoprogenitor cells [36].

Further, van Hengel *et al.* developed additively manufactured titanium implants with reduced risk of implant-associated infection through the incorporation silver nanoparticles in an oxide surface layer coating [37]. In general, for bone tissue engineering applications, metals are an attractive category of material due to their excellent mechanical properties and structural stability. However, many metals have a Young’s modulus much higher than that of natural bone that can lead to stress shielding and resorption of the surrounding bone tissue [10]. A common solution to reduce the stiffness of metallic implants is to increase the porosity, which simultaneously improves the possibility of vascularization within the scaffold. For example, He *et al.* demonstrated that increasing the porosity of a titanium scaffold from 44.2% to 65.1% leads to a decrease in the elastic modulus from 1.22 to 0.18 GPa, thereby approaching the elastic modulus of cancellous bone [38].

However, there are remaining limitations to the use of metallic scaffolds including non-degradability, fatigue, ion-release and risk of infection. In addition, metallic scaffolds continue to demonstrate a lack of integration with host tissue and often lead to the formation of fibrous tissue which poses a threat to long-term scaffolds success [29]. The non-degradability of metals is the main drawback limiting its use as scaffold materials. Indeed, metallic scaffolds will not degrade over time as cells invade the scaffold pores forming new bone tissue, creating problems associated with the long-term presence of metals within the tissue. One solution includes the fabrication of magnesium alloy-based highly porous scaffolds, which can degrade *in vivo* by corrosion. However, despite the fact that magnesium degradation has been shown to stimulate bone-healing, concerns remain regarding the inflammatory response to the degradation of metals *in vivo* as well as the body’s ability to clear the corrosion products [24].

#### Ceramics

Similar to metals, ceramics have been used commonly as biomaterials for orthopedic applications, both in the form of ceramic implants and coatings for implant fixation. However, ceramics are formed into highly porous structures by methods such as selective laser sintering and 3D printing in order to be used as bone tissue engineering scaffolds. Calcium phosphates (CaPs), particularly hydroxyapatite (HAp) and tricalcium phosphate (TCP) are among the most widely used bone substitute materials due to their compositional similarity to bone mineral and excellent chemical biocompatibility [29, 39].

Hydroxyapatite is an osteoconductive material, presenting a porosity similar to that of native bone and thereby promoting the growth of bone tissue along the surface or within the pores of the scaffold [40]. In addition, the calcium and phosphate ions released during HAp degradation induce an osteogenic response, contributing to the osteoinductivity of these materials [3]. In addition, TCP and HAp present no immunogenicity or toxic side effects [19].

Ceramics present high compressive strength close to that of trabecular bone [4] and can be formed into highly interconnected macroporous structures, thereby promoting vascularization, nutrient delivery and bone ingrowth [33, 41, 42]. Additional key advantages of CaP-based scaffolds include their versatility and tailorable biodegradability when compared to other ceramics [29]. However, the clinical application of ceramic-based scaffolds is limited by their poor performance in load-bearing applications.

Despite the toughness of ceramics, their highly brittle nature is a key limiting factor in the regeneration of loaded bone [27]. One solution has been the development of ceramic-based composite scaffolds. Such systems combine the excellent biocompatibility of calcium phosphates with the durability of biocompatible polymers, the advantages of which are outlined in the following section.

#### Polymers

**Figure 2.**
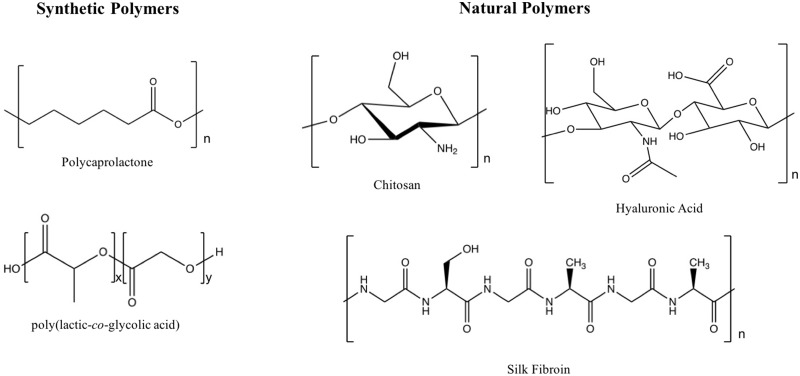
Structures of common synthetic and natural polymers used for bone tissue engineering

Polymeric scaffolds have emerged as excellent candidates for bone tissue regeneration, primarily due to their versatile and tunable properties (Fig. 2). A key advantage of biodegradable polymers, specifically, is their ability to support tissue regeneration and remodeling before being resorbed by the body [20].

##### Synthetic polymers

Key advantages of synthetic polymers lie in the ability to tune properties. In fabricating scaffolds from synthetic polymers, it is possible to control degradation rate, to tune mechanical properties and to fabricate complex shapes [4]. In addition, synthetic polymers are highly reliable source materials [43]. Common synthetic polymers for bone tissue engineering include poly(glycolic acid), poly(lactic acid), copolymers of poly(DL-lactic–glycolic acid) (PLGA), polycaprolactone (PCL) and many others [44]. Synthetic polymers are especially attractive in the context of hydrogels, which are highly hydrated polymeric networks and therefore attractive materials to be used in tissue engineering and drug delivery systems [45].

Despite the versatility provided by organic polymer synthesis, the lack of bioactivity of the resulting material restricts interactions with the host tissue [46]. This is especially apparent when compared to the bioactivity of natural polymers, which uniquely present ECM binding domains and thereby improved tissue integration. One solution to address the limited bioactivity of synthetic polymers has been to fabricate composite materials. For example, Kumar *et al.* [45] prepared a hybrid hydrogel composed of polyacrylamide, sodium alginate and silica glass, reinforced with cellulose nanocrystals (CNCs). The authors showed that the incorporation of CNCs improved scaffold mechanical performance. In addition, the hybrid hydrogels demonstrated good *in vitro* apatite-forming ability and the presence of CNCs led to better *in vitro* osteoblast cytocompatibility.

An additional limitation of synthetic polymers is the adverse tissue response to acidic degradation or toxic degradation byproducts [29, 44]. As an example of properties that limit utility in bone regeneration, PLLA scaffolds are hydrophobic, lack homogeneous incorporation of proteins and achieve poor cell attachment [47]. These limitations have led researchers to explore the possibility of using natural polymers for bone regeneration.

##### Natural polymers

Among the most common natural polymers used for bone tissue engineering applications are collagen, silk fibroin, chitosan, as well as alginate and hyaluronic acid due to their superior chemical biocompatibility, low immunogenicity and proven ability to facilitate cell growth [44]. In addition, natural polymer scaffold porosity, charge and mechanical strength can be tuned by optimizing polymer concentration and fabrication conditions [1]. Natural polymers also present a range of ligands that have been shown to facilitate bone cell adhesion [39].

Natural polymer-based porous scaffolds can be fabricated via a wide range of methods including fiber bonding, melt molding, solvent casting, gas foaming, phase separation and electrospinning [1]. In addition, natural polymers are used to fabricate hydrogels. These 3D networks are characterized by tissue-like water content, structure stability and homogeneous cell encapsulation leading to improved biocompatibility [48]. In addition, the soft and bendable characteristics of hydrogels minimize damage to surrounding tissue [49]. Some limitations of natural polymers are their difficulty in controlling their degradation rate as well as low mechanical stability [4].

Collagen is a widely used material for bone tissue engineering because collagen I is abundant in bone tissue [39]. Collagen hydrogels are inherently chemically biocompatible and biodegradable, highly porous, minimally antigenic and can easily be combined with other materials [32]. Collagen plays a key role in promoting the osteogenic differentiation of bone progenitor cells via alpha beta integrin receptor interactions, thereby promoting cell growth and mineral production [39]. Schneider *et al.* evaluated the response of both bone marrow and umbilical cord-derived mesenchymal stem cells (MSC) to a 3D collagen I/III matrix [50]. The collagen scaffold’s ability to promote MSC adhesion, migration into the scaffold, growth, spreading, osteogenic differentiation and ECM degradation and synthesis were evaluated. It was shown that in response to the collagen matrix, both bone marrow-derived MSCs and umbilical cord-derived MSCs displayed key features of functional osteoblasts including osteogenic gene expression, ECM mineralization, and the ability to colonize the scaffold, which are required for proper fracture healing. A limitation of collagen-based scaffolds is the relatively poor mechanical properties, though hybrid scaffolds incorporating collagen combine the excellent biocompatibility of collagen with the improved mechanical properties of another material. Some additional limitations of include high manufacturing costs and high *in vivo* swelling due to the high hydrophilicity of collagen [32].

In addition to collagen, silk fibroin is an attractive natural polymer for bone tissue engineering applications. Indeed, silk fibroin-based materials offer excellent mechanical properties, biocompatibility and versatility in processing [33]. Silk fibroin is environmentally stable, flexible and degradable by proteolytic enzymes [51]. Finally, silk fibroin scaffolds support the osteogenic differentiation of MSCs and preserve protein bioactivity [52]. Kim *et al.* developed a biomimetic scaffold by incorporating the bone-like mineral hydroxyapatite into a highly porous silk fibroin network for a GF-free approach [33]. The extent of osteoconductivity was assessed based on the *in vitro* response of hMSCs. Premineralization of the silk fibroin scaffolds led to increased alkaline phosphatase activity and calcium deposition and therefore increased osteogenic outcomes.

Finally, another natural polymer commonly used for bone scaffolds is chitosan. Chitosan is the deacetylated form of chitin, which is an abundant natural resource, most commonly sourced not only from skeletal materials of crustaceans but also present in mushroom envelopes, green algae cell walls and yeast [49]. Multiple reports indicate the superior ability of chitosan to promote cell adhesion and proliferation as well as osteoblast differentiation when compared to other natural and synthetic polymers [1].

Chitosan has also been shown to promote osteoconductivity, enhance bone mineralization [53] and present antibacterial, analgesic, hemostatic and mucoadhesive properties [43, 54]. The structural properties of chitosan impart some of its key attractive properties. Chitosan degradation *in vivo* occurs through the breaking of glyosidic bonds by lysozyme [49, 55], forming non-toxic oligosaccharides [1]. The degree of deacetylation as well as the molecular mass determine the degradation rate of chitosan [49]. In addition, the presence of a protonable amino groups results in its mucoadhesive properties while the positive charges on the chitosan backbone impart the natural polymer’s hemostatic properties [1]. The ability of chitosan to open tight junction proteins by interacting with the negative part of the cell membrane also results in its permeation-enhancing properties [49].

Chitosan hydrogels can be formed by physical association, coordination complex crosslinking and chemical crosslinking [49]. In general, the presence of primary amines and secondary hydroxyl groups on the chitosan backbone facilitates the addition of side groups [55]. A limitation in the use of chitosan is, similar to other natural polymers, the inferior mechanical strength of chitosan scaffolds when compared to metal, ceramic and synthetic polymer-based networks.

However, Jana *et al.* fabricated high strength 3D chitosan scaffolds for bone tissue engineering applications [31]. Scaffolds were fabricated by increasing chitosan concentrations from 4 to 12 wt%. Scaffolds fabricated from 12 wt% chitosan solutions where characterized by a porosity of 86.1% with a pore wall thickness of 45 μm and pores ranging from 100 to 500 μm in size. These superior strength scaffolds had a compressive strength of 1.74 MPa and a Young’s Modulus of 1.28 MPa. In addition, increased mechanical strength led to improved adhesion, proliferation and osteogenic activity of MG-63 osteoblasts. The mechanical properties can also be improved through the incorporation of higher strength synthetic polymers resulting in a hybrid scaffold. Saber-Samandari developed a chitosan-graft-poly(acrylic acid-co-acrylamide)/hydroxyapatite scaffold using a freeze drying method [56]. A weight ratio of chitosan/hydroxyapatite of 100:25 led to the formation of scaffolds with an average pore size of 108 μm, a compressive modulus of 2.15 MPa and an elastic modulus of 0.33 GPa, resembling the properties of trabecular bone that has a compressive strength and elastic modulus of 5.30 MPa and 0.44 GPa, respectively [57].

## Incorporation and delivery of GFs

Bone tissue relies on the action of GFs that provide signals at the injury site thereby allowing progenitors and inflammatory cells to migrate and initiate the healing process [44]. These GFs instruct cell behavior through specific binding to transmembrane receptors on the target cells. In addition, GFs do not act in an endocrine fashion, but rather by short range diffusion through the ECM [58]. It is therefore believed that, in addition to mimicking the fibrillar structure of the ECM, scaffolds should be equipped with bioactive molecules such as GFs, angiogenic factors, differentiation factors or drugs [14].

Early GF delivery approaches for tissue regeneration, such as direct injection or systematic local supplementation, resulted in low availability of bioactive GFs due to their rapid degradation *in vivo*, short half-life in physiological conditions, and deactivation by enzymes [16, 59]. The poor pharmacokinetics of these proteins has led to the delivery of supraphysiological doses, which has increased the risk of adverse effects [60].

Indeed, it has been shown that supraphysiological doses of bone-related GFs can result in the formation of heterotopic bone, pseudoarthrosis, local inflammation and immune response [61]. The development of delivery vehicles, which allow for the controlled and sustained release of GFs, has potential to reduce the need for large doses and the occurrence of side effects. The aim of GF delivery in tissue engineering is therefore to increase the occurrence of healing, limit excessive bone formation, accelerate the healing process and generally improve the delivery of therapeutics [62]. The following section of this review will focus on the incorporation of GFs into scaffolds for bone tissue engineering applications.

### GFs for bone regeneration

**Figure 3.**
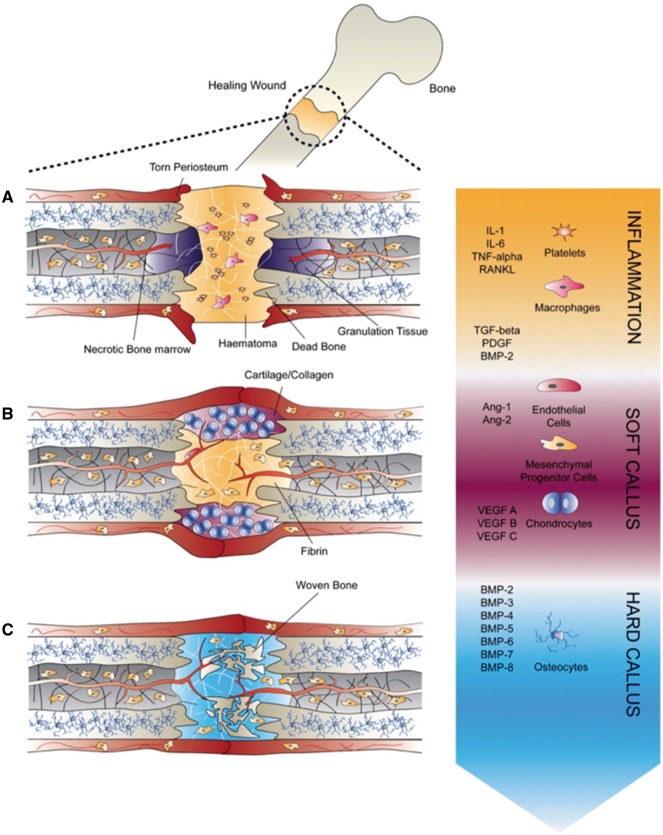
Bone fracture healing steps. A spatiotemporal cascade of GFs regulates the regeneration of bone during fracture repair. The healing process can be categorized into three stages: (A) inflammatory phase, (B) soft callus formation during which angiogenesis occurs ensuring the vascularization of the newly formed bone and (C) hard callus formation during which osteogenic GFs promote the differentiation of recruited mesenchymal progenitor cells. Figure reproduced from Lienemann *et al.* [7]

Bone fracture healing is a multi-step process orchestrated by a complex spatiotemporal cytokine cascade, requires the combination of various cell types including inflammatory cells, vascular cells, mesenchymal progenitor cells and osteocytes (Fig. 3) [7, 9]. These signaling cascades result from the elevated expression of different pro-inflammatory, angiogenic and osteogenic GFs [63]. Tissue engineering approaches therefore aim to combine cells and engineering materials with these signaling biomolecules crucial for effective tissue repair [64]. The main families of GFs involved in bone regeneration include fibroblast GFs (FGF), bone morphogenetic proteins (BMPs), vascular endothelial GF (VEGF), insulin-like GF (IGF) and transforming GF β (TGFβ) [16]. Particular attention has been directed toward the use of BMP-2 and BMP-7, which have been incorporated in Food and drug Administration (FDA)-approved devices for bone regeneration. The GFs used in bone tissue engineering can be classified as inflammatory GFs and cytokines, pro-osteogenic GFs and angiogenic GFs, and their individual roles in bone fracture repair are outlined in the following sections.

#### Inflammatory factors

Inflammation is the first stage in bone fracture repair [65] and occurs during the first days after fracture occurs [66]. Vascular disruption upon injury leads to the formation of a clot at the bone fracture site, and an inflammatory response is initiated. Inflammatory cells are recruited to the clot through multiple pro-inflammatory signaling molecules, which are released by platelets *in vivo* [65]. Indeed, the key role of inflammatory cytokines is to promote the invasion of lymphocytes, plasma cells, macrophages and osteoclasts [9]. The key inflammatory cytokines include tumor necrosis factor α, which increases osteoclast activity, FGF-2, interleukin-1 (IL-1), IL-6 and macrophage colony-stimulating factor [7].

#### Angiogenic factors

A lack of vascularization, or ischemia, is one of the primary risk factors for reduced bone healing. Vessels not only provide oxygen but also are a conduit for additional osteoblasts, play a positive-signaling role for promoting cell differentiation and are required for endochondral ossification [65].

Angiogenesis is defined as the formation of new vessels from a pre-existing vascular network and is considered to be an essential process for proper bone regeneration as it provides the necessary nutritional support for the newly formed tissue as well as a cell source for further tissue remodeling [62]. The key pro-angiogenic factors identified include platelet-derived GF (PDGF), BMPs, FGFs and TGFβ [7].

FGFs are involved in the FGF pathway, which induces angiogenesis by promoting the proliferation of endothelial and osteoblast cells [6]. FGF-2 (or bFGF) plays a key role in angiogenesis, wound healing, and tissue repair, though a key challenge to its delivery is its very short half-live *in vivo* of 90s pointing to the need for carrier methods to extend its action time [67].

VEGF is a key regulator of angiogenesis during bone formation, with VEGF expression peaking during the early days after bone fracture. After fracture, low oxygen tensions occur and are sensed by hypoxia inducible factor, which targets the transcription of VEGF and in turn promotes neovascularization [9, 65]. Indeed, VEGF stimulates the proliferation and migration of endothelial cells leading to the formation of tubular blood vessels and subsequently promotes the recruitment and survival of bone forming cells [65, 68]. However, VEGF induces vascular permeability, which can lead to systemic hypotension and edema [68], therefore requiring adequate control of quantity and rate of VEGF delivery. In order to demonstrate the importance of VEGF in bone tissue regeneration, Kempen *et al.* developed a system for the sequential release of VEGF with BMP-2, a key regulator of osteogenesis [13]. The *in vivo* performance of BMP-2-loaded PLGA microspheres in a poly(propylene fumarate) (PPF) scaffold combined with a VEGF-loaded gelatin hydrogel in a rat subcutaneous model demonstrated both improved vessel and bone formation when compared to scaffolds that did not contain VEGF.

#### Osteogenic factors

Bone repair relies on the recruitment of progenitors, which can differentiate into bone-forming osteoblasts. Bone is then formed through endochondral and intramembranous ossification and subsequently remodeled by osteoclasts for the formation of new intramembranous bone [65]. A key role of osteoblasts is the deposition of organic ECM components such as fibrillary proteins, glycoproteins, sialoproteins and proteoglycans with glycosaminoglycans (GAGs) [7]. These GAG components play a critical role through their interaction with GFs critical for morphogenetic processes. Multiple pro-osteogenic GFs have been identified including PDGF, TGFβ, FGF, IGF and BMPs [7]. Among these, BMP signaling is the most widely understood pathway for bone regeneration.

The most well-characterized members of the BMP family include BMP-2, BMP-4 and BMP-7 [65]. In fact, BMP-2 and BMP-7 have been used clinically to treat open tibia fracture, non-union bone injuries and spinal fusion and are incorporated in FDA-approved systems for bone regeneration [69]. The use of these GFs is therefore a promising strategy for improving bone tissue engineering approaches.

BMPs play an important role in initiation the fracture repair cascade, and primarily act by triggering osteogenic differentiation of osteoprogenitors and MSCs recruited to the injury site [6, 70]. In particular, BMP-2 is considered to be the most notable cytokine and plays a key role in the expression of osteogenic markers [59]. Specifically, alkaline phosphatase (ALP) and osteocalcin expression are early indicators of osteogenesis and are increased by the action of BMP-2 [71].

In addition to BMPs, other GFs have been identified as potent signaling molecules to further promote bone formation. IGF-1 is released when osteoclasts resorb fractured bone matrix [9] and acts as a mitogenic factor stimulating the growth and differentiation of embryonic cells which then stimulate osteoblast growth and proliferation [59]. Stromal-derived factor (SDF-1) is a key factor in the recruitment and migration of stem cells in the early fracture repair stages and leads to new bone more densely populated with MSCs [52, 65]. Similarly, PDGF leads to an increase in bone formation and is one of the primary initiating signals for cellular ingress into the fracture site [65]. Finally, bFGF in addition to stimulating angiogenesis can promote an increase in the number of osteocytes and is a potent mitogen for MSCs.

### GF carrier requirements

As evidenced by the wide range of inflammatory, angiogenic and osteogenic factors involved, bone tissue repair is a highly dynamic process that relies on cellular and biomolecular responses over periods of several weeks [8]. In order to meet their therapeutic roles in an effective manner, GFs must reach the injury site without loss of bioactivity and remain in the target location over the therapeutic time frame [72]. This prompts the need for release technologies capable of the controlled spatiotemporal delivery of signaling molecules for bone tissue regeneration.

An efficient GF carrier must therefore control GF release kinetics in order to optimize tissue formation. Release profiles could include extended release, multifactorial release or sequential release, depending on the GFs to be delivered, and their biological requirements [39]. Specifically, for the delivery of BMP-2, an initial burst release followed by a slow, sustained release over several weeks leads to improved bone regeneration [9]. An optimal carrier for GFs must also allow site-specific delivery and promote enhanced infiltration of cells [63]. In addition, the carrier must load each GF efficiently, promote robust carrier-protein assembly [72], and encourage the presentation of proteins to cell surface receptors [53]. Finally, the carrier fabrication process should be simple, feasible and preserve the bioactivity of the incorporated protein. Beyond sustained release of single GFs, the delivery of multiple GFs with biologically inspired temporal, spatial and dosing parameters is a key strategy for improved bone tissue engineering [46]. Since a challenge in bone tissue engineering lies in the regeneration of vascularized bone tissue, one solution has been the co-delivery of osteogenic and angiogenesis-promoting GFs [73]. In general, the delivery of multiple GFs enhances biomimicry of the natural bone-healing process [69].

Ultimately, the aim of scaffold-based GF delivery is to precisely coordinate the cell response by matching biological signaling to the physiological dynamics of bone fracture repair [46]. In addition to scaffold material selection and choice of appropriate GFs, the design of a system that delivers physiologically relevant doses of GF in a targeted manner and preserves their bioactivity for prolonged periods is a key strategy in improving bone tissue engineering technologies.

### GF delivery approaches

In order to meet the requirements for GF delivery, several scaffold-based strategies have been explored. GF delivery methods can rely on the physical entrapment of proteins within the scaffold, the covalent or non-covalent binding of the proteins to the scaffold, or the use of micro- or nanoparticles as protein reservoirs (Table 2). It should be noted that a wide range of delivery systems have been developed, based on a variety of materials and to deliver a wide variety of GFs. The present review aims to provide a brief overview of some recent examples in the literature and the principles behind these approaches. A selection of these delivery systems is outlined in Table 3.

**Table 2.** Schematic representations of the reviewed incorporation strategies and their resulting growth factor release profiles

Incorporation strategy	Schematic	Release profile [72]	Advantages (+) and limitations (−)
Covalent binding	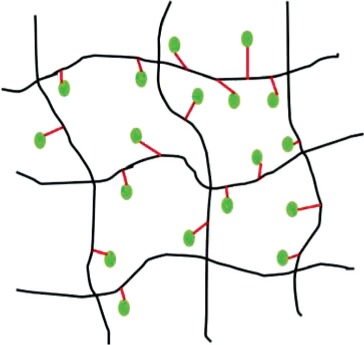	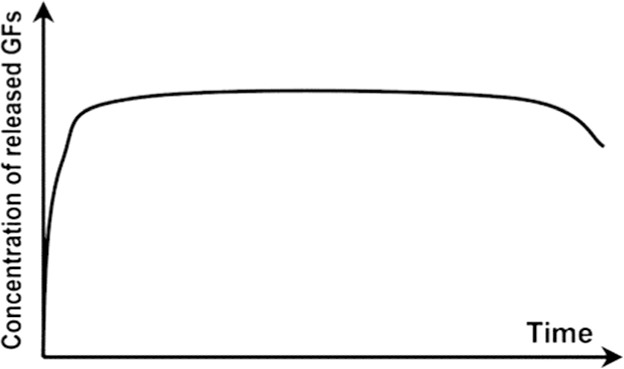	+ No GF diffusion out of scaffold − Loss of GF bioactivity
Physical entrapment/ Adsorption	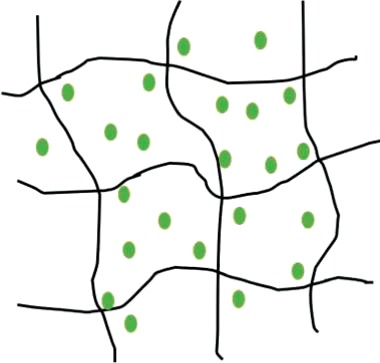	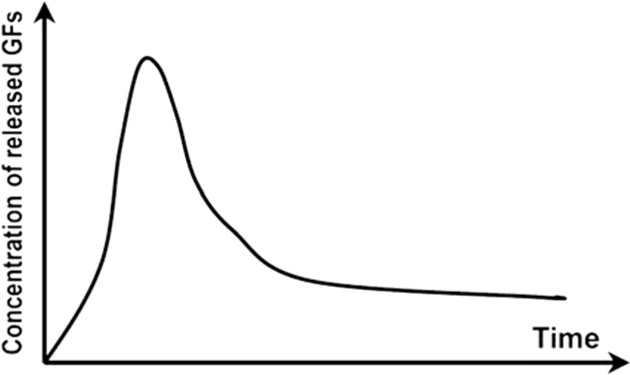	+ GF bioactivity maintained − Diffusion and degradation mediated release − Rapid burst release − Difficult to control release rate
Incorporation into micro/nanospheres	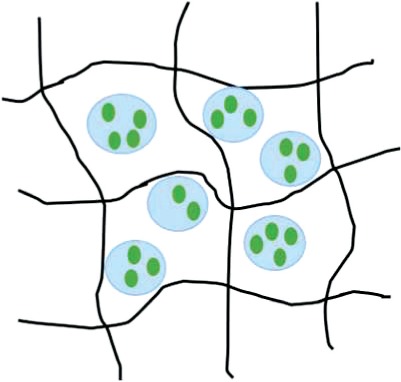	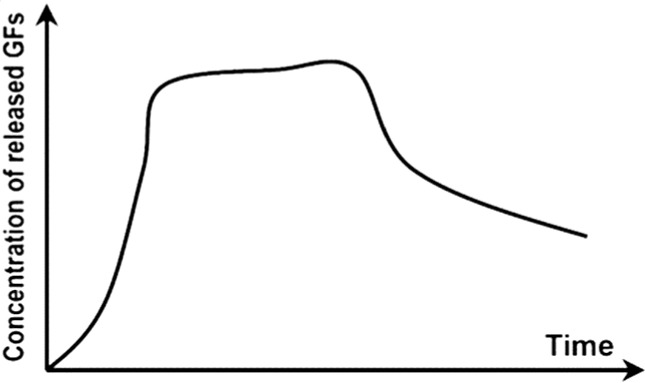	+ Better control of GF release rate + Possibility for sequential release of multiple GFs − Additional fabrication steps − Can rely on use of harsh chemicals

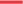
 = covalent bond; 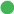 = growth factor; 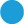 = micro/nanocapsule.

**Table 3.** Selected growth factor delivery systems for bone tissue engineering applications

GF(s)	Incorporation approach	Carrier material	Release profile	Results	References
GDF-5	Physical entrapment	Collagen membrane	Sustained release for 21 days	*In vitro*: increased ALP activity and osteocalcin expression of MC3T3-E1 cells	[74]
				*In vivo*: enhanced bone regeneration in rat mandibular defect model	
	Physical entrapment	Hyaluronic acid hydrogel	Initial burst release then sustained for 28 days	*In vitro*: improved MC3T3-E1 differentiation and proliferation	[75]
				*In vivo*: improved osteogenesis in a rabbit bone defect model	
BMP-2	Adsorption	Polyelectrolyte multilayer film coating on PLGA tube	Rapid burst release	*In vivo*: complete defect bridging, formation of vascularized and mineralized bone in rat critical-size defect model	[65]
	Adsorption	Chitosan layers on electrospun PCL fibers	N/A	*In vitro*: enhanced osteopontin expression and mineralization of scaffolds	[14]
				*In vivo*: improved CaP mineralization in murine calvarial defect model	
	Physical entrapment	Silica xerogel-chitosan coating on porous HAp scaffold	Sustained release up to 6 weeks	*In vitro*: improved osteoblastic differentiation	[41]
				*In vivo*: improved bone regeneration and interaction with host tissue in rabbit calvarial defect model	
	Nanoparticle encapsulation	Chitosan/chondroitin sulfate nanoparticles in biphasic calcium phosphate scaffolds	Initial burst release over 6 days, sustained release for 6 weeks	*In vitro*: improved osteoconductivity	[42]
				*In vivo*: improved mineralization of ectopic bone in rabbits	
	Nanoparticle encapsulation	Nanoporous silica nanoparticles	N/A	*In vitro*: increased ALP activity, osteocalcin production, bone nodule formation in adMSCs	[76]
	Nanoparticle encapsulation	PLL nanoparticles in fibrin hydrogel	Reduced burst effect followed by high release rate	*In vitro*: enhanced osteogenic differentiation of hMSCs	[48]
BMP-2 and VEGF	Microparticle encapsulation	Gelatin microparticles in PPF scaffold	Burst release in first 24h, sustained release for 27 days	*In vivo*: no increase in bone formation with addition of VEGF in rat cranial critical size defect	[77]
	Physical entrapment/ microparticle encapsulation	PLGA microspheres in gelatin hydrogel	Burst release of VEGF in first 3 days, sustained release of BMP-2 for 56 days	*In vivo*: VEGF enhanced BMP-2-induced ectopic bone formation	[13]
	Nano-/microparticle encapsulation	PLGA nanoparticles, alginate microcapsules in collagen scaffold	Burst release in first 3 days, sustained release up to 28 days, BMP-2 released at higher concentration	*In vitro*: improved osteogenesis of umbilical cord blood-derived (UCB)-MSCs	[73]
				*In vivo*: improved bone formation and vascularization in rat calvarial defect	
BMP-2 and IGF-1	Adsorption	Gelatin coating	Burst release of BMP-2 followed by sustained delivery of BMP-2 and IGF-1 after 5 days	*In vitro*: increased ALP activity, matrix mineralization in C3H cultures	[78]
	Adsorption/microparticle encapsulation	Gelatin microspheres in chitosan scaffold	Initial release of BMP-2 followed by slow, sustained release of IGF-1 for 1 week	*In vitro*: increased ALP activity in W-20-17 stromal cells	[59]
BMP-2 and GDF-5	Nanoparticle encapsulation	Thermoresponsive elastin nanoparticles	Initial burst release for 24h, gradual release for 14 days	*In vitro*: increased ALP activity and mineralization in C2C12 cells	[70]
BMP-2 and TGFb	Physical entrapment	Alginate hydrogel	N/A	*In vivo*: ectopic bone formation by bMSCs 6 weeks after implantation in mice	[79]
	Covalent binding	PCL-POEGMA scaffolds	No release of GFs from scaffold	*In vitro*: enhanced osteochondral differentiation of hMSCs	[80]
BMP-2 and SDF-1	Adsorption/nanoparticle encapsulation	Silk fibroin microspheres in HAp scaffold	Rapid initial release of SDF-1 in first days, slow sustained release of BMP-2 for three weeks	*In vitro*: improved recruitment and osteogenic differentiation of bMSCs	[52]
				*In vivo*: enhanced bone regeneration 12 weeks post implantation in rat calvarial critical size defect	
BMP-2 and BMP-7	Nanoparticle encapsulation	PLGA and PHBV nanocapsules in chitosan fiber mesh	No burst release, sustained release for 25 days, higher release rate from PLGA than PHBV	*In vitro*: sequential delivery of BMP-2 followed by BMP-7 led to increased ALP activity of bMSCs	[81]
VEGF and PDGF	Physical entrapment/microparticle encapsulation	Alginate microspheres in chitosan hydrogel	Initial burst release followed by sustained release. PDGF released more rapidly than VEGF	*In vivo*: enhanced bone formation in rabbit femoral defect	[82]

#### Covalent attachment to scaffold

One method for the incorporation of GFs into scaffolds is the chemical reacting of proteins to polymer matrices. This requires the modification of GFs to contain reactive functional groups such as thiols, acrylates, azides and Gln tags [7]. This method generally reduces burst release and allows for a prolonged release of the GF that was bound to the carrier [58]. Release kinetics in this case are mediated by hydrolysis and reduction reactions, or by cell-mediated enzymatic cleavage [64]. He *et al.* investigated the effect of covalently binding RGD and BMP peptides to a hydrogel substrate on the osteogenic differentiation and mineralization of bone marrow stromal cells (bMSCs) [83]. RGD sequences, which are associated with ECM proteins, interact with bMSCs through integrin surface receptors and promote cell adhesion and spreading. A poly(lactide-co-ethylene oxide fumarate) (PLEOF) hydrogel was fabricated by crosslinking, with an acrylamide-terminated RDG peptide. An azide functionalized, PEGylated BMP peptide was then conjugated to the PLEOF hydrogel by click chemistry. In response to these peptide functionalized hydrogels, bMSCs increased their ALP expression and calcium content, suggesting the synergistic action of RGD and BMP peptides toward osteogenic differentiation.

Di Luca *et al.* explored covalent binding of GFs to additively manufactured 3D scaffolds [80]. Additively manufactured poly(ε-caprolactone) scaffolds were modified with functionalizable poly(oligo (ethylene glycol) methacrylate) (POEGMA) brushes, which were subsequently functionalized with BMP-2 and TGF-β3. The brush-supported GFs significantly affected hMSCs osteogenic and chondrogenic differentiation.

However, covalent binding of proteins is time intensive, labor intensive and costly. In addition, due to the difficulty in controlling the modification site, covalent binding may block active sites on the protein, thereby interfering with GF bioactivity [7]. Covalent binding can also be used for the surface immobilization of proteins to the scaffold, by introducing reactive functional groups on the surface of the scaffold. This method, while able to improve protein-loading efficiency, still risks modifying protein conformation and therefore reducing protein bioactivity [83].

#### Non-covalent binding to scaffold

Non-covalent binding of GFs to scaffolds relies on the physical entrapment or bulk incorporation of the protein within the 3D matrix. GFs become immobilized within the scaffold through mechanisms such as physical encapsulation, protein adsorption or the formation of ionic complexes [84]. Protein adsorption is often considered to be the simplest method of GF delivery and is the method used by current commercially available GF delivery systems [84]. Bioactive molecules are incorporated by immersing the preformed scaffold in a protein solution [47]. Protein adsorption to scaffolds can be controlled by varying certain material properties such as surface wettability, roughness, surface charge, charge density and the presence of functional groups [17].

Non-covalent protein incorporation into scaffolds leads to the delivery of GFs in an initial, uncontrolled burst release. The mechanisms of release include protein desorption, scaffold degradation and a failure of the protein to interact with the scaffold [17]. GF delivery from non-covalently bound systems is therefore both a diffusion and degradation-dependent process [64]. The diffusion-dependent release follows a first-order release profile that is dependent on the size of the GF relative to the scaffold pore size [66]. Diffusion is limited when the scaffold pores are smaller than the hydrodynamic radius of the incorporated protein [64]. In this case, GF release is governed by surface and/or bulk erosion of the scaffold. The delivery of GFs then becomes linked to the spatiotemporal degradation profile of the encapsulating matrix [85].

Key limitations of non-covalent protein adsorption to scaffolds are the loading efficiency and poor control of release kinetics [84]. These systems are characterized by a burst release of the incorporated GFs, followed by a degradation-mediated release dependent on the degradation mechanism of the scaffold material. A certain degree of control over release rate is therefore possible by altering the material degradation [7]. Efforts to improve loading efficiency have focused on increasing the electrostatic attraction between GFs such as BMP-2 and the scaffold matrix [17].

A bioinspired approach to the incorporation of GFs into scaffolds is through the incorporation of naturally derived components involved in receptor-ligand interactions [86]. Indeed, components such as heparin, fibronectin, gelatin and hyaluronic acid provide specific biological sites for GF immobilization [58]. The key advantage of these affinity-based systems is that no harsh chemicals are required during the protein encapsulation process, as the scaffolds are chemically modified prior to incorporating the desired GFs. For example, Steffens *et al.* covalently incorporated heparin into a collagen matrix using zero length crosslinking mediated by EDC/NHS [87]. Physical immobilization of VEGF within this ligand-containing scaffold increased the material’s angiogenic potential. Kim *et al.* developed PCL/PLGA scaffolds via solid freeform fabrication and conjugated heparin and dopamine to control the delivery of BMP-2 [34]. These scaffolds exhibited a burst release followed by sustained release of BMP-2 for 28 days. These scaffolds led to enhanced osteoblast activity *in vitro* as well as bone formation and mineralization *in vivo* in a rat femur defect.

Different experimental methods have been applied for the non-covalent incorporation of GFs including electrospinning, hydrogel incorporation and polyelectrolyte multilayer film coating. Sahoo *et al.* evaluated the potential of two different electrospinning techniques as methods to incorporate GFs into ECM mimicking electrospun nanofibrous scaffolds [67]. bFGF-releasing PLGA nanofibers were fabricated by blending and electrospinning, leading to random dispersion of GF, as well as by coaxial electrospinning, which leads to a central core of bFGF within the nanofibers. It was found that electrospinning with the protein included in the solution leads to the continuous release of bFGF over a shorter time than from the coaxially electrospun fibers. GFs have also been incorporated into scaffolds by simply including the GF suspension in the polymer solution during material fabrication. Chang *et al.* evaluated the ability of porous PLLA scaffolds to act as carriers for rhBMP-2 [47]. The PLLA scaffolds were fabricated by freeze drying and freeze extraction, and rhBMP-2 was incorporated by adding the GF solution to the polymer solution before freezing. The *in vivo* results demonstrated that the incorporation of rhBMP-2 not only alleviated the adverse effects of PLLA but also led to improved bone formation in the rat ectopic bone formation model.

GFs have also been loaded into scaffolds by adsorption from an aqueous solution. Draenert *et al.* evaluated the loading and release of BMP-2 and bFGF onto different biological scaffolds and their effect on an *in vitro* bone model [84]. The GFs were diluted in phosphate buffered saline and then applied to the different scaffold materials that were dried. The scaffolds were washed in PBS and vortexed in order to remove non-adsorbed GFs. For all scaffold materials, a sustained release was observed, though differences in release kinetics existed depending on the chosen material and GF used.

The use of hydrogels is also common in GF delivery strategies as they can act both as scaffolding materials and as protein-releasing matrices [64]. For example, Bae *et al.* developed photo-cured hyaluronic acid (HA) hydrogels loaded with growth and differentiation factor 5 (GDF-5), which is a member of the BMP family and an important factor in limb development [75]. 2-Aminoethyl methacrylate (AEMA)-conjugated HA hydrogels were fabricated by irradiating the mixture of HA-AEMA and a photoinitiator. GDF-5 was incorporated into the hydrogel by mixing the GF solution with HA-AEMA prior to crosslinking. The hydrogels demonstrated an initial burst release followed by sustained release for high loading concentrations over 28 days. The release of GDF-5 led to expression of osteogenic markers *in vitro* as well as the *in vivo* bone formation in a rabbit bone defect model.

Finally, non-covalent GF delivery with a polyelectrolyte film has also been explored. Bouyer *et al.* developed a PLGA scaffold with a polyelectrolyte film surface coating as a carrier for BMP-2 [85]. The polyelectrolyte multilayer film coating was deposited using PLL and HA, and crosslinked using EDC/NHS, and BMP-2 was post-loaded into the polyelectrolyte multilayer films. The material was tested *in vivo* on a critical-size rat femoral defect and exhibited rapid bone healing for 1-2 weeks.

#### Nanoparticle incorporation into scaffold

The final strategy for GF delivery in bone tissue engineering applications is through incorporating nanoparticles into the bone tissue engineering scaffold. Indeed, delivery systems relying on non-covalent protein incorporation rely on the adsorption of the protein into the biomaterial and its slow desorption at the local site. The encapsulation of proteins within nanoparticles, which are then delivered by scaffolds would allow for more precise control of their release and achieve the long-term sustained release profiles desired for certain GFs and applications [42, 88].

GF-encapsulating particles can be synthesized using a wide range of methods including solvent evaporation, precipitation, single and double emulsion and electrospraying [43]. Although it is among the most common methods, the use of organic solvents in the emulsion method presents the risk of denaturing the loaded proteins. Methods like electrospraying, in which an electric field is applied and the resulting electrostatic forces counteract the liquid surface tension leading to the formation of small liquid droplets, could be a simple technique for nanoparticle fabrication [43]. Wang *et al.* employed a polyelectrolyte complexation process to prepare chitosan/chondroitin sulfate nanoparticles containing BMP-2 [42]. This method does not require the use of harsh organic solvents or high temperatures and can thereby retain protein bioactivity. These nanoparticles were then immobilized on biphasic calcium phosphate scaffolds and the *in vitro* response of bMSCs as well as *in vivo* ectopic bone formation in rats were evaluated. It was demonstrated that the polysaccharide nanoparticles as BMP-2 carriers were effective in improving the osteoconductivity of the scaffold.

The incorporation of nanoparticles into scaffolds as protein carriers presents multiple advantages. First, nanotechnology has been used in bone tissue engineering to overcome limitations associated with poor mechanical properties [43]. Indeed, nanoparticle incorporation into scaffolds has proven to improve the bulk mechanical strength of scaffolds [89].

Nanoparticles and nanotopology in general improve osteointegration, osteoconduction and osteoinduction by mimicking the complex hierarchical structures of the natural bone environment and providing a favorable milieu for cell attachment and ingrowth [59, 90]. Other advantages of nanoparticles include their small size, large specific surface area, and high drug-loading efficiency [60]. Encapsulating proteins within nanocarriers also ensures their protection from enzymes *in vivo* and allows for prolonged protein retention [63]. The rate of GF release is highly dependent on particle size, so it is possible to obtain a certain degree of control over the protein release profiles [91].

Suliman *et al.* [91] evaluated the effect of different BMP-2 immobilization techniques on the GF release and bioactivity. BMP-2 was (i) adsorbed onto poly(LLA-co-CL) scaffolds, (ii) adsorbed onto scaffolds modified with nanodiamond particles, (iii) covalently linked to nanodiamond particles or (iv) encapsulated in PLGA microspheres. The results of *in vitro* and *in vivo* studies suggested that adsorption onto scaffolds does not promote consistent osteogenic potential, whereas the incorporation into microspheres resulted in gradual and sustained increase in GF release, as well as consistent satisfactory bone formation.

Nano- and microparticle incorporation also give rise to the possibility of multimodal delivery of one or multiple GFs, by, for example, embedding GF-loaded microspheres into a hydrogel structure, which itself acts as a protein carrier [64, 67]. A sequential GF delivery system was developed by Yilgor *et al.* [81] based on the incorporation of PLGA and PHBV nanocapsules into wet-spun chitosan scaffolds. PLGA and PHGV nanocapsules loaded with BMP-2 and BMP-7, respectively, were fabricated by double emulsion-solvent evaporation. These nanoparticles were incorporated into the wet spun chitosan scaffolds both by incorporating within the fibers, and by loading onto the fibers. In vitro testing of the response of bone marrow-derived MSCs demonstrated that the use of different nanocapsules allowed for the sequential delivery of BMP-2 followed by BMP-7 which in turn led to an increase in ALP activity.

Shen *et al.* [52] developed a system for the sequential release of SDF-1 and BMP-2 from a silk fibroin-nano-hydroxyapatite (SF/nHAp) scaffold. BMP-2 was loaded into silk fibroin microspheres via a laminar jet break up method, which were in turn incorporated into the SF/nHAp scaffold, while SDF-1 was incorporated directly into the scaffold via physical adsorption. The *in vitro* release profiles indicated an initial rapid burst release of SDF-1, followed by a prolonged release of BMP-2 for up to 3 weeks. Both *in vitro* and *in vivo* results indicated enhanced bone regeneration when compared to single GF delivery. This system thereby mimics the natural bone healing process, with early stage stem cell migration promoted by SDF-1 followed by the activation of osteoblastic differentiation by BMP-2 in the later stages.

Subbiah *et al.* [73] developed a dual GF delivery system of BMP-2 and VEGF to promote both angiogenesis and osteogenesis. BMP-2-loaded PLGA nanoparticles were produced using an ultrasonication-assisted double emulsion evaporation method. These nanoparticles were then incorporated in VEGF-encapsulating alginate microcapsulses via electrodropping. These microcapsules were then incorporated into a 3D collagen disc. The performance of these scaffolds in an *in vivo* rat calvarial defect model reveal the synergistic effect of the dual delivery system in the development of vascularized bone tissue. These composite systems can also be used for the co-delivery of GFs and drugs which stimulate bone formation.

Gan *et al.* proposed a dual-delivery system for the delivery of BMP-2 and dexamethasone (Dex), which is a glucocorticoid proven to stimulate BMP-2-induced osteoblast differentiation [53]. This system relies on the adsorption of BMP-2 onto a hydrophilic chitosan coating on mesoporous silica nanoparticles (MSN), which contain Dex within their hydrophobic nanochannels. The BMP-2 readily dissociates from the chitosan coating in physiological conditions while Dex remains within the MSN channels. After endocytosis into cells, a decrease in pH results in a response of chitosan and the release of Dex into the cytosol. In vitro results demonstrated that BMP-2 and Dex resulted in osteogenic effects both outside and within the cell simultaneously.

An advantage in using polymeric nanoparticles is the high degree of versatility through the functionalizability of the polymer chains [90]. Park *et al.* developed a fibrin hydrogel containing PLL nanoparticles for the delivery of BMP-2 [48]. The PLL nanoparticles were heparinized and loaded with BMP-2, and then mixed with a solution of fibrinogen and hMSCs for the preparation of the fibrin hydrogel scaffolds. The incorporation of BMP-2 into nanoparticles led to a reduction in the initial burst release, improved retention of BMP-2 bioactivity after 21 days and improved osteogenic differentiation *in vitro*. Finally, nanoparticles themselves can be used as osteoinductive factors with bioglass and hydroxyapatite nanoparticles having demonstrated bone inducing capabilities [90].

## Conclusions and future challenges

Bone tissue engineering research has significantly advanced, in terms of scaffold fabrication and in promising strategies for GF delivery. This review first outlined the biological, mechanical and structural requirements in the design of a successful scaffold, followed by an overview of common materials for scaffold fabrication. The different GFs involved in inflammation, angiogenesis and osteogenesis, which are the key phases in bone fracture repair, were then summarized. Subsequently, the various requirements for optimal GF delivery systems were described. Finally, this review provided a brief overview of some of the key GF delivery approaches existing in the literature, namely, the covalent binding of GFs to scaffolds, non-covalent immobilization of GFs within scaffolds and encapsulation of GFs within scaffold-incorporated nanoparticles. The use of nanoparticles, in particular, has provided promising results.

Despite these findings, bone tissue engineering continues to face multiple challenges to clinical implementation. First, the field must optimize and precisely control scaffold physical and mechanical properties. More work is required to develop highly porous and strong biomaterials, with controlled biodegradation, matching the rate of new bone formation. Some promising directions of research include the fabrication of composite scaffolds, combining the mechanical properties of bone-like ceramics with the degradability and osteogenicity of natural polymers such as collagen, chitosan or hyaluronic acid. Another approach with great potential is the incorporation of nanoparticles, both to increase osteogenicity by incorporating nanotopography, as well as to improve mechanical properties. This nanotechnology-based approach could also address the difficulty of introducing the micro- and nanoscale dimensional hierarchy representative of bone.

Another challenge facing the widespread implementation of bone tissue engineering approaches is the difficulty of regenerating properly vascularized bone tissue. One approach to promote vascularization is the presence of an interconnected network of large pores within the scaffold. However, vascularization can be further improved through incorporating and releasing bioactive molecules with controlled kinetics. In fact, GF delivery has become the focus of bone tissue engineering research in recent years and goes beyond the aim of promoting angiogenesis. The incorporation of osteogenic GFs is considered to be essential for the regeneration of functional bone tissue. The greatest focus has been on developing and improving methods for the controlled, long-term delivery of BMP-2, which is considered to be the most potent osteogenic GF.

Due to the known local and systemic side effects of direct GF injection such as edema, hypotension, hypoplasia, there is a clinical need for delivery systems that provide precise spatiotemporal control. Understanding the parameters that affect the delivery rates of GFs will be essential in developing systems that allow their controlled delivery. Recent focuses include incorporating nanoparticles, which encapsulate GFs within scaffolds, and have shown promising results. Achieving this level of control could lead to the design of sequential release systems with the ultimate goal of mimicking the coordinated fracture repair pathway. The delivery of multiple GFs in a sequential and targeted manner could not only promote the essential phases of bone formation: inflammation, angiogenesis and osteogenesis but also form gradient structures within the newly formed bone tissue, such as the osteochondral interface.

Ultimately, further efforts both in terms of scaffold design and protein delivery strategies will be essential in order to move toward the clinical implementation of bone tissue engineering. These technologies present a promising solution to address the growing occurrence and economic impact of bone-related injuries, and have the potential to alleviate the high costs and negative side effects of the over 1.6 million annual bone grafting procedures.

## References

[1] Balagangadharan K, Dhivya S, Selvamurugan N. Chitosan based nanofibers in bone tissue engineering. Int J Biol Macromol 2017;104:1372–82. 27993655 10.1016/j.ijbiomac.2016.12.046

[2] Porter JR, Ruckh TT, Popat KC. Bone tissue engineering: a review in bone biomimetics and drug delivery strategies. Biotechnol Prog 2009;25:1539–60. 19824042 10.1002/btpr.246

[3] Amini AR, Laurencin CT, Nukavarapu SP. Bone tissue engineering: recent advances and challenges. Crit Rev Biomed Eng 2012;40:363–408. 23339648 10.1615/critrevbiomedeng.v40.i5.10 PMC3766369

[4] Roseti L, Parisi V, Petretta M et al Scaffolds for bone tissue engineering: state of the art and new perspectives. Mater Sci Eng C 2017;78:1246–62. 10.1016/j.msec.2017.05.017 28575964

[5] Habibovic P. Strategic directions in osteoinduction and biomimetics. Tissue Eng Part A 2017;23:1295–6. 29032745 10.1089/ten.TEA.2017.0430

[6] Bose S, Vahabzadeh S, Bandyopadhyay A. Bone tissue engineering using 3D printing. Mater Today 2013;16:496–504.

[7] Lienemann PS, Lutolf MP, Ehrbar M. Biomimetic hydrogels for controlled biomolecule delivery to augment bone regeneration. Adv Drug Deliv Rev 2012;64:1078–89. 22465487 10.1016/j.addr.2012.03.010

[8] Hettiaratchi MH, Rouse T, Chou C et al Enhanced in vivo retention of low dose BMP-2 via heparin microparticle delivery does not accelerate bone healing in a critically sized femoral defect. Acta Biomater 2017;59:21–32. 28645809 10.1016/j.actbio.2017.06.028 PMC6546418

[9] Nyberg E, Holmes C, Witham T et al Growth factor-eluting technologies for bone tissue engineering. Drug Deliv Transl Res 2016;6:184–94. 25967594 10.1007/s13346-015-0233-3

[10] Wu S, Liu X, Yeung KWK et al Biomimetic porous scaffolds for bone tissue engineering. Mater Sci Eng R Rep 2014;80:1–36.

[11] Martin V, Bettencourt A. Bone regeneration: biomaterials as local delivery systems with improved osteoinductive properties. Mater Sci Eng C 2018;82:363–71. 10.1016/j.msec.2017.04.038 29025670

[12] Delloye C, Cornu O, Druez V et al Bone allografts: what they can offer and what they cannot. Bone Jt J 2007;89:574–80. 10.1302/0301-620X.89B5.19039 17540738

[13] Kempen DHR, Lu L, Heijink A et al Effect of local sequential VEGF and BMP-2 delivery on ectopic and orthotopic bone regeneration. Biomaterials 2009;30:2816–25. 19232714 10.1016/j.biomaterials.2009.01.031

[14] Ferrand A, Eap S, Richert L et al Osteogenetic properties of electrospun nanofibrous PCL scaffolds equipped with chitosan-based nanoreservoirs of growth factors. Macromol Biosci 2014;14:45–55. 23956214 10.1002/mabi.201300283

[15] Loh QL, Choong C. Three-dimensional scaffolds for tissue engineering applications: role of porosity and pore size. Tissue Eng Part B Rev 2013;19:485–502. 23672709 10.1089/ten.teb.2012.0437 PMC3826579

[16] Azevedo HS, Pashkuleva I. Biomimetic supramolecular designs for the controlled release of growth factors in bone regeneration. Adv Drug Deliv Rev 2015;94:63–76. 26325686 10.1016/j.addr.2015.08.003

[17] King WJ, Krebsbach PH. Growth factor delivery: how surface interactions modulate release in vitro and in vivo. Adv Drug Deliv Rev 2012;64:1239–56. 22433783 10.1016/j.addr.2012.03.004 PMC3586795

[18] Hankenson KD, Zimmerman G, Marcucio R. Biological perspectives of delayed fracture healing. Injury 2014;45:S8–15. 24857030 10.1016/j.injury.2014.04.003 PMC4406220

[19] Kolk A, Handschel J, Drescher W et al Current trends and future perspectives of bone substitute materials – from space holders to innovative biomaterials. J Craniomaxillofac Surg 2012;40:706–18. 22297272 10.1016/j.jcms.2012.01.002

[20] Liu Y, Lim J, Teoh S-H. Review: development of clinically relevant scaffolds for vascularised bone tissue engineering. Biotechnol Adv 2013;31:688–705. 23142624 10.1016/j.biotechadv.2012.10.003

[21] Janbaz S, Noordzij N, Widyaratih DS et al Origami lattices with free-form surface ornaments. Sci Adv 2017;3:eaao1595. 29209661 10.1126/sciadv.aao1595 PMC5710187

[22] Tamaddon M, Samizadeh S, Wang L et al intrinsic osteoinductivity of porous titanium scaffold for bone tissue engineering. Int J Biomater 2017;2017:1. DOI: 10.1155/2017/5093063. 10.1155/2017/5093063 PMC5549492 28814954

[23] Dabrowski B, Swieszkowski W, Godlinski D et al Highly porous titanium scaffolds for orthopaedic applications. J Biomed Mater Res B Appl Biomater 2010;95:53–61. 20690174 10.1002/jbm.b.31682

[24] Witte F, Ulrich H, Palm C et al Biodegradable magnesium scaffolds: part II: peri-implant bone remodeling. J Biomed Mater Res A 2007;81:757–65. 17390322 10.1002/jbm.a.31293

[25] Balla VK, Bodhak S, Bose S et al Porous tantalum structures for bone implants: fabrication, mechanical and in vitro biological properties. Acta Biomater 2010;6:3349–59. 20132912 10.1016/j.actbio.2010.01.046 PMC2883027

[26] Tiainen H, Lyngstadaas SP, Ellingsen JE et al Ultra-porous titanium oxide scaffold with high compressive strength. J Mater Sci Mater Med 2010;21:2783–92. 20711636 10.1007/s10856-010-4142-1 PMC2962783

[27] Fu Q, Saiz E, Rahaman MN et al Bioactive glass scaffolds for bone tissue engineering: state of the art and future perspectives. Mater Sci Eng C 2011;31:1245–56. 10.1016/j.msec.2011.04.022 PMC3169803 21912447

[28] Seitz H, Rieder W, Irsen S et al Three-dimensional printing of porous ceramic scaffolds for bone tissue engineering. J Biomed Mater Res B Appl Biomater 2005;74:782–8. 15981173 10.1002/jbm.b.30291

[29] Bose S, Tarafder S. Calcium phosphate ceramic systems in growth factor and drug delivery for bone tissue engineering: a review. Acta Biomater 2012;8:1401–21. 22127225 10.1016/j.actbio.2011.11.017 PMC3418064

[30] Panseri S, Cunha C, D'Alessandro T et al Magnetic hydroxyapatite bone substitutes to enhance tissue regeneration: evaluation in vitro using osteoblast-like cells and in vivo in a bone defect. PLoS One 2012;7:e38710. 22685602 10.1371/journal.pone.0038710 PMC3369900

[31] Jana S, Florczyk SJ, Leung M et al High-strength pristine porous chitosan scaffolds for tissue engineering. J Mater Chem 2012;22:6291.

[32] Ferreira AM, Gentile P, Chiono V et al Collagen for bone tissue regeneration. Acta Biomater 2012;8:3191–200. 22705634 10.1016/j.actbio.2012.06.014

[33] Kim HJ, Kim U-J, Kim HS et al Bone tissue engineering with premineralized silk scaffolds. Bone 2008;42:1226–34. 18387349 10.1016/j.bone.2008.02.007 PMC2698959

[34] Kim T-H, Yun Y-P, Park Y-E et al In vitro and in vivo evaluation of bone formation using solid freeform fabrication-based bone morphogenic protein-2 releasing PCL/PLGA scaffolds. Biomed Mater 2014;9:025008. 24518200 10.1088/1748-6041/9/2/025008

[35] Grafahrend D, Heffels K-H, Beer MV et al Degradable polyester scaffolds with controlled surface chemistry combining minimal protein adsorption with specific bioactivation. Nat Mater 2011;10:67–73. 21151163 10.1038/nmat2904

[36] Van Bael S, Chai YC, Truscello S et al The effect of pore geometry on the in vitro biological behavior of human periosteum-derived cells seeded on selective laser-melted Ti6Al4V bone scaffolds. Acta Biomater 2012;8:2824–34. 22487930 10.1016/j.actbio.2012.04.001

[37] van Hengel IAJ, Riool M, Fratila-Apachitei LE et al Selective laser melting porous metallic implants with immobilized silver nanoparticles kill and prevent biofilm formation by methicillin-resistant *Staphylococcus aureus*. Biomaterials 2017;140:1–15. 28622569 10.1016/j.biomaterials.2017.02.030

[38] He G, Liu P, Tan Q. Porous titanium materials with entangled wire structure for load-bearing biomedical applications. J Mech Behav Biomed Mater 2012;5:16–31. 22100076 10.1016/j.jmbbm.2011.09.016

[39] Fernandez-Yague MA, Abbah SA, McNamara L et al Biomimetic approaches in bone tissue engineering: integrating biological and physicomechanical strategies. Adv Drug Deliv Rev 2015;84:1–29. 25236302 10.1016/j.addr.2014.09.005

[40] Lu J, Yu H, Chen C. Biological properties of calcium phosphate biomaterials for bone repair: a review. RSC Adv 2018;8:2015–33. 10.1039/c7ra11278e PMC9077253 35542623

[41] Jun S-H, Lee E-J, Jang T-S et al Bone morphogenic protein-2 (BMP-2) loaded hybrid coating on porous hydroxyapatite scaffolds for bone tissue engineering. J Mater Sci Mater Med 2013;24:773–82. 23344924 10.1007/s10856-012-4822-0

[42] Wang Z, Wang K, Lu X et al BMP-2 encapsulated polysaccharide nanoparticle modified biphasic calcium phosphate scaffolds for bone tissue regeneration. J Biomed Mater Res A 2015;103:1520–32. 25100662 10.1002/jbm.a.35282

[43] Jayaraman P, Gandhimathi C, Venugopal JR et al Controlled release of drugs in electrosprayed nanoparticles for bone tissue engineering. Adv Drug Deliv Rev 2015;94:77–95. 26415888 10.1016/j.addr.2015.09.007

[44] Mouriño V, Boccaccini AR. Bone tissue engineering therapeutics: controlled drug delivery in three-dimensional scaffolds. J R Soc Interface 2010;7:209–27. 19864265 10.1098/rsif.2009.0379 PMC2842615

[45] Kumar A, Rao KM, Han SS. Synthesis of mechanically stiff and bioactive hybrid hydrogels for bone tissue engineering applications. Chem Eng J 2017;317:119–31.

[46] Shrivats AR, McDermott MC, Hollinger JO. Bone tissue engineering: state of the union. Drug Discov Today 2014;19:781–6. 24768619 10.1016/j.drudis.2014.04.010

[47] Chang P-C, Liu B-Y, Liu C-M et al Bone tissue engineering with novel rhBMP2-PLLA composite scaffolds. J Biomed Mater Res A 2007;81:771–80. 17226806 10.1002/jbm.a.31031

[48] Park K-H, Kim H, Moon S et al Bone morphogenic protein-2 (BMP-2) loaded nanoparticles mixed with human mesenchymal stem cell in fibrin hydrogel for bone tissue engineering. J Biosci Bioeng 2009;108:530–7. 19914589 10.1016/j.jbiosc.2009.05.021

[49] Croisier F, Jérôme C. Chitosan-based biomaterials for tissue engineering. Eur Polym J 2013;49:780–92.

[50] Schneider RK, Puellen A, Kramann R et al The osteogenic differentiation of adult bone marrow and perinatal umbilical mesenchymal stem cells and matrix remodelling in three-dimensional collagen scaffolds. Biomaterials 2010;31:467–80. 19815272 10.1016/j.biomaterials.2009.09.059

[51] Polo-Corrales L, Latorre-Esteves M, Ramirez-Vick JE. Scaffold design for bone regeneration. J Nanosci Nanotechnol 2014;14:15–56. 24730250 10.1166/jnn.2014.9127 PMC3997175

[52] Shen X, Zhang Y, Gu Y et al Sequential and sustained release of SDF-1 and BMP-2 from silk fibroin-nanohydroxyapatite scaffold for the enhancement of bone regeneration. Biomaterials 2016;106:205–16. 27566869 10.1016/j.biomaterials.2016.08.023

[53] Gan Q, Zhu J, Yuan Y et al A dual-delivery system of pH-responsive chitosan-functionalized mesoporous silica nanoparticles bearing BMP-2 and dexamethasone for enhanced bone regeneration. J Mater Chem B 2015;3:2056–66. 10.1039/c4tb01897d 32262373

[54] Venkatesan J, Anil S, Kim S-K et al Chitosan as a vehicle for growth factor delivery: various preparations and their applications in bone tissue regeneration. Int J Biol Macromol 2017;104:1383–97. 28109812 10.1016/j.ijbiomac.2017.01.072

[55] Saravanan S, Leena RS, Selvamurugan N. Chitosan based biocomposite scaffolds for bone tissue engineering. Int J Biol Macromol 2016;93:1354–65. 26845481 10.1016/j.ijbiomac.2016.01.112

[56] Saber-Samandari S, Saber-Samandari S. Biocompatible nanocomposite scaffolds based on copolymer-grafted chitosan for bone tissue engineering with drug delivery capability. Mater Sci Eng C 2017;75:721–32. 10.1016/j.msec.2017.02.112 28415522

[57] Karageorgiou V, Kaplan D. Porosity of 3D biomaterial scaffolds and osteogenesis. Biomaterials 2005;26:5474–91. 15860204 10.1016/j.biomaterials.2005.02.002

[58] Lee K, Silva EA, Mooney DJ. Growth factor delivery-based tissue engineering: general approaches and a review of recent developments. J R Soc Interface 2011;8:153–70. 20719768 10.1098/rsif.2010.0223 PMC3033020

[59] Kim S, Kang Y, Krueger CA et al Sequential delivery of BMP-2 and IGF-1 using a chitosan gel with gelatin microspheres enhances early osteoblastic differentiation. Acta Biomater 2012;8:1768–77. 22293583 10.1016/j.actbio.2012.01.009 PMC3314097

[60] Walmsley GG, McArdle A, Tevlin R et al Nanotechnology in bone tissue engineering. Nanomedicine Nanotechnol Biol Med 2015;11:1253–63. 10.1016/j.nano.2015.02.013 PMC4476906 25791811

[61] Visser R, Rico-Llanos GA, Pulkkinen H et al Peptides for bone tissue engineering. J Controlled Release 2016;244:122–35. 10.1016/j.jconrel.2016.10.024 27794492

[62] Varkey M, Gittens SA, Uludag H. Growth factor delivery for bone tissue repair: an update. Expert Opin Drug Deliv 2004;1:19–36. 16296718 10.1517/17425247.1.1.19

[63] Vo TN, Kasper FK, Mikos AG. Strategies for controlled delivery of growth factors and cells for bone regeneration. Adv Drug Deliv Rev 2012;64:1292–309. 22342771 10.1016/j.addr.2012.01.016 PMC3358582

[64] Censi R, Di Martino P, Vermonden T et al Hydrogels for protein delivery in tissue engineering. J Controlled Release 2012;161:680–92. 10.1016/j.jconrel.2012.03.002 22421425

[65] Hankenson KD, Gagne K, Shaughnessy M. Extracellular signaling molecules to promote fracture healing and bone regeneration. Adv Drug Deliv Rev 2015;94:3–12. 26428617 10.1016/j.addr.2015.09.008

[66] Newman MR, Benoit DS. Local and targeted drug delivery for bone regeneration. Curr Opin Biotechnol 2016;40:125–32. 27064433 10.1016/j.copbio.2016.02.029 PMC4975663

[67] Sahoo S, Ang LT, Goh JC-H et al Growth factor delivery through electrospun nanofibers in scaffolds for tissue engineering applications. J Biomed Mater Res A 2010;93A:1539–50. 10.1002/jbm.a.32645 20014288

[68] Martino MM, Briquez PS, Maruyama K et al Extracellular matrix-inspired growth factor delivery systems for bone regeneration. Adv Drug Deliv Rev 2015;94:41–52. 25895621 10.1016/j.addr.2015.04.007

[69] Farokhi M, Mottaghitalab F, Shokrgozar MA et al Importance of dual delivery systems for bone tissue engineering. J Controlled Release 2016;225:152–69. 10.1016/j.jconrel.2016.01.033 26805518

[70] Bessa PC, Machado R, Nürnberger S et al Thermoresponsive self-assembled elastin-based nanoparticles for delivery of BMPs. J Controlled Release 2010;142:312–8. 10.1016/j.jconrel.2009.11.003 19913578

[71] Sivashankari PR, Prabaharan M. Prospects of chitosan-based scaffolds for growth factor release in tissue engineering. Int J Biol Macromol 2016;93:1382–9. 26899174 10.1016/j.ijbiomac.2016.02.043

[72] Chen F-M, Zhang M, Wu Z-F. Toward delivery of multiple growth factors in tissue engineering. Biomaterials 2010;31:6279–308. 20493521 10.1016/j.biomaterials.2010.04.053

[73] Subbiah R, Hwang MP, Van SY et al Osteogenic/angiogenic dual growth factor delivery microcapsules for regeneration of vascularized bone tissue. Adv Healthc Mater 2015;4:1982–92. 26138344 10.1002/adhm.201500341

[74] Yamano S, Haku K, Yamanaka T et al The effect of a bioactive collagen membrane releasing PDGF or GDF-5 on bone regeneration. Biomaterials 2014;35:2446–53. 24388383 10.1016/j.biomaterials.2013.12.006

[75] Bae MS, Ohe J-Y, Lee JB et al Photo-cured hyaluronic acid-based hydrogels containing growth and differentiation factor 5 (GDF-5) for bone tissue regeneration. Bone 2014;59:189–98. 24291420 10.1016/j.bone.2013.11.019

[76] Neumann A, Christel A, Kasper C et al BMP2-loaded nanoporous silica nanoparticles promote osteogenic differentiation of human mesenchymal stem cells. RSC Adv 2013;3:24222–30.

[77] Young S, Patel ZS, Kretlow JD et al Dose effect of dual delivery of vascular endothelial growth factor and bone morphogenetic protein-2 on bone regeneration in a rat critical-size defect model. Tissue Eng Part New Rochelle 2009;15:2347–62. 10.1089/ten.tea.2008.0510 PMC2792218 19249918

[78] Raiche AT, Puleo DA. In vitro effects of combined and sequential delivery of two bone growth factors. Biomaterials 2004;25:677–85. 14607506 10.1016/s0142-9612(03)00564-7

[79] Simmons CA, Alsberg E, Hsiong S et al Dual growth factor delivery and controlled scaffold degradation enhance in vivo bone formation by transplanted bone marrow stromal cells. Bone 2004;35:562–9. 15268909 10.1016/j.bone.2004.02.027

[80] Di Luca A, Klein-Gunnewiek M, Vancso JG et al Covalent binding of bone morphogenetic protein-2 and transforming growth factor-β3 to 3D plotted scaffolds for osteochondral tissue regeneration. Biotechnol J 2017;12: doi: 10.1002/biot.201700072. 10.1002/biot.201700072 28865136

[81] Yilgor P, Tuzlakoglu K, Reis RL et al Incorporation of a sequential BMP-2/BMP-7 delivery system into chitosan-based scaffolds for bone tissue engineering. Biomaterials 2009;30:3551–9. 19361857 10.1016/j.biomaterials.2009.03.024

[82] De la Riva B, Sánchez E, Hernández A et al Local controlled release of VEGF and PDGF from a combined brushite–chitosan system enhances bone regeneration. J Controlled Release 2010;143:45–52. 10.1016/j.jconrel.2009.11.026 19963026

[83] He X, Ma J, Jabbari E. Effect of grafting RGD and BMP-2 protein-derived peptides to a hydrogel substrate on osteogenic differentiation of marrow stromal cells. Langmuir 2008;24:12508–16. 18837524 10.1021/la802447v

[84] Draenert FG, Nonnenmacher A-L, Kämmerer PW et al BMP-2 and bFGF release and in vitro effect on human osteoblasts after adsorption to bone grafts and biomaterials. Clin Oral Implants Res 2013;24:750–7. 22524399 10.1111/j.1600-0501.2012.02481.x

[85] Bouyer M, Guillot R, Lavaud J et al Surface delivery of tunable doses of BMP-2 from an adaptable polymeric scaffold induces volumetric bone regeneration. Biomaterials 2016;104:168–81. 27454063 10.1016/j.biomaterials.2016.06.001 PMC5937675

[86] Lee SS, Huang BJ, Kaltz SR et al Bone regeneration with low dose BMP-2 amplified by biomimetic supramolecular nanofibers within collagen scaffolds. Biomaterials 2013;34:452–9. 23099062 10.1016/j.biomaterials.2012.10.005 PMC3496771

[87] Steffens GCM, Yao C, Prével P et al Modulation of angiogenic potential of collagen matrices by covalent incorporation of heparin and loading with vascular endothelial growth factor. Tissue Eng Larchmt 2004;10:1502–9. 10.1089/ten.2004.10.1502 15588409

[88] Zhang S, Wang G, Lin X et al Polyethylenimine-coated albumin nanoparticles for BMP-2 delivery. Biotechnol Prog 2008;24:945–56. 19194903 10.1002/btpr.12

[89] Corona-Gomez J, Chen X, Yang Q. Effect of nanoparticle incorporation and surface coating on mechanical properties of bone scaffolds: a brief review. J Funct Biomater 2016;7:18. DOI: 10.3390/jfb7030018. 10.3390/jfb7030018 PMC5040991 27420104

[90] Vieira S, Vial S, Reis RL et al Nanoparticles for bone tissue engineering. Biotechnol Prog 2017;33:590–611. 28371447 10.1002/btpr.2469

[91] Suliman S, Xing Z, Wu X et al Release and bioactivity of bone morphogenetic protein-2 are affected by scaffold binding techniques in vitro and in vivo. J Controlled Release 2015;197:148–57. 10.1016/j.jconrel.2014.11.003 25445698

